# Seasonal upsurge of pneumococcal meningitis in the Central African Republic

**DOI:** 10.12688/wellcomeopenres.14868.2

**Published:** 2019-03-29

**Authors:** Thomas Crellen, V. Bhargavi Rao, Turid Piening, Joke Zeydner, M. Ruby Siddiqui

**Affiliations:** 1 Department of Mathematical and Economic Modelling, Mahidol-Oxford Tropical Medicine Research Unit, Bangkok, 10400, Thailand; 2 Médecins Sans Frontières Operational Centre Amsterdam, Bangui, Boite Postale 1793, Central African Republic; 3 Médecins Sans Frontières, London, EC4A 1AB, UK; 4 Médecins Sans Frontières, Berlin, 10179, Germany

**Keywords:** Central African Republic, pneumococcal meningitis, Streptococcus pneumoniae, Neisseria meningitidis, meningococcal meningitis, African meningitis belt

## Abstract

A high incidence of bacterial meningitis was observed in the Central African Republic (CAR) from December 2015 to May 2017 in three hospitals in the northwest of the country that are within the African meningitis belt. The majority of cases were caused by *Streptococcus pneumoniae* (249/328; 75.9%), which occurred disproportionately during the dry season (November-April) with a high case-fatality ratio of 41.6% (95% confidence interval [CI] 33.0, 50.8%). High rates of bacterial meningitis during the dry season in the meningitis belt have typically been caused by *Neisseria meningitidis* (meningococcal meningitis), and our observations suggest that the risk of contracting *S. pneumoniae* (pneumococcal) meningitis is increased by the same environmental factors. Cases of meningococcal meningitis (67/328; 20.4%) observed over the same period were predominantly group W and had a lower case fatality rate of 9.6% (95% CI 3.6, 21.8%). Due to conflict and difficulties in accessing medical facilities, it is likely that the reported cases represented only a small proportion of the overall burden. Nationwide vaccination campaigns in the CAR against meningitis have been limited to the use of MenAfriVac, which targets only meningococcal meningitis group A. We therefore highlight the need for expanded vaccine coverage to prevent additional causes of seasonal outbreaks.

## Introduction

The northern districts of the Central African Republic (CAR) form part of the meningitis belt, a broad swathe of sub-Saharan Africa where the incidence of bacterial meningitis typically peaks during the dry season ^1^. The mechanism for the increase in cases of bacterial meningitis has been postulated to be a result of damage to host mucosal defenses by the extreme environmental conditions of the dry season in this region, resulting in an increased rate of conversion from asymptomatic carriage to invasive disease ^2^. The CAR is among the world’s least developed nations and large areas of the country remain unstable following conflict between ethnic groups in 2013 and 2014. Consequently reliable medical data is scarce.

While outbreaks in the meningitis belt, including the CAR, have historically been caused by meningococcus group A ^3, 4^, national vaccination campaigns with MenAfriVac, which targets exclusively this group, has resulted in a shift in the epidemiology of bacterial meningitis. A surveillance study in ten African countries found that since MenAfriVac was introduced in certain countries in 2010, the proportion of confirmed cases due to meningococcus group A dropped substantially, while the proportion attributable to meningococcus group W and pneumococcus increased over the same period ^5^. The CAR began nationwide vaccination with MenAfriVac in 2016, though coverage levels have not been assessed.

**Figure 1.**
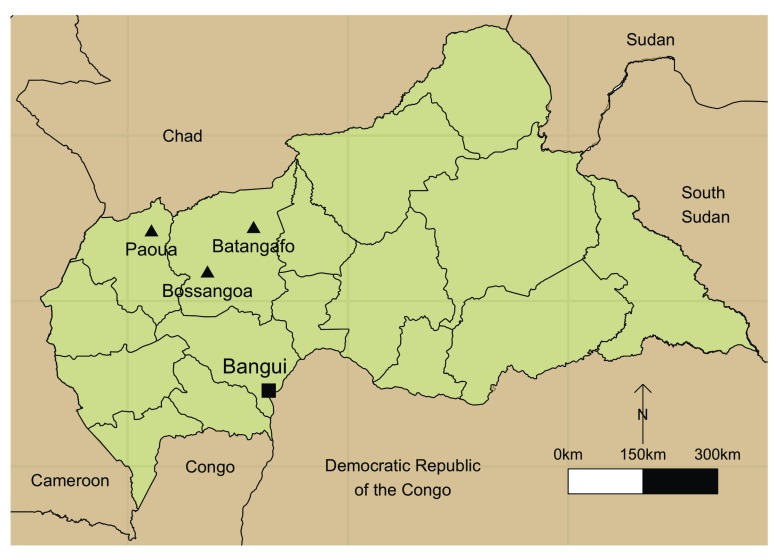
Map showing the location of the three reporting hospitals in the Central African Republic: Bossangoa, Batangafo and Paoua in relation to one another and to the capital Bangui.

Here we report on findings from routinely collected data on meningitis patients in health facilities supported by the medical organisation Médecins Sans Frontières (MSF) in Bossangoa, Ouham Prefecture from 1st December 2015 to 31st May 2017, along with additional data from MSF supported hospitals in Batangafo, Ouham Prefecture and Paoua, Ouham-Pendé Prefecture within the same period. These towns are all located in the northwest of CAR close to the Chadian border and are separated by linear distances of 130–200km (see map, Figure 1).

The country is shown divided by prefecture (highest administrative level), Bossangoa and Batangafo are located in Ouham prefecture and Paoua is located in Ouham-Pendé prefecture.

## Methods

### Patients

Data were collected prospectively from three reporting hospitals (shown in Figure 1) from December 2015 to May 2017 as part of routine communicable disease surveillance. Our confirmed case criteria and clinical management of patients followed the guidelines from the World Health Organization ^6^. Our analysis included all confirmed cases with bacterial meningitis in the three hospitals over the observed periods. Data on suspected and negative cases were collected in Paoua using established definitions ^5^. Patient outcomes were recorded in Bossangoa and Batangafo along with socio-demographic characteristics (age, sex, residence) in all regions. Patient outcomes and incidence rates have previously been reported from Paoua Hospital over a subset of our observation period ^7^.

### Laboratory Testing

Bacterial meningitis cases were confirmed by a latex agglutination test (Pastorex, Bio-Rad; cat. No. 61607) on cerebrospinal fluid (CSF), following the guidelines of the CAR Ministry of Health (MoH). The causative agent was confirmed by PCR of CSF samples at the Pasteur Institute in Bangui for around 5% of patients. PCR conditions were identical to those given by Corless *et al*. ^8^.

### Statistical analysis

Patient data were recorded using Microsoft Excel and statistical analysis was performed in R (version 3.5.1). Logistic regression and a chi-squared test for counts were performed. Statistical significance was defined as *p*<0.05 or non-overlapping 95% confidence intervals.

### Ethical Statement

Médecins Sans Frontières (MSF) is able to operate in countries such as the Central African Republic only with the support of national and local authorities and through continued dialogue with the beneficiary communities.

The high incidence of meningitis cases during this period was known to the communities and MSF gave health advice and provided medical services during the outbreak. Communities in the CAR were made aware that MSF collects data as part of its medical operations, including for research purposes. This research fulfilled the exemption criteria set by the MSF Ethical Review Board (ERB) for *a posteriori* analyses of routinely collected clinical data and thus did not require MSF ERB review. Data collected from patients was anonymized, though patient consent was not sought retrospectively. This study was conducted with permission from the Medical Director Sidney Wong (MSF-Operational Centre Amsterdam).

## Results

### Overview of cases

**Figure 2.**
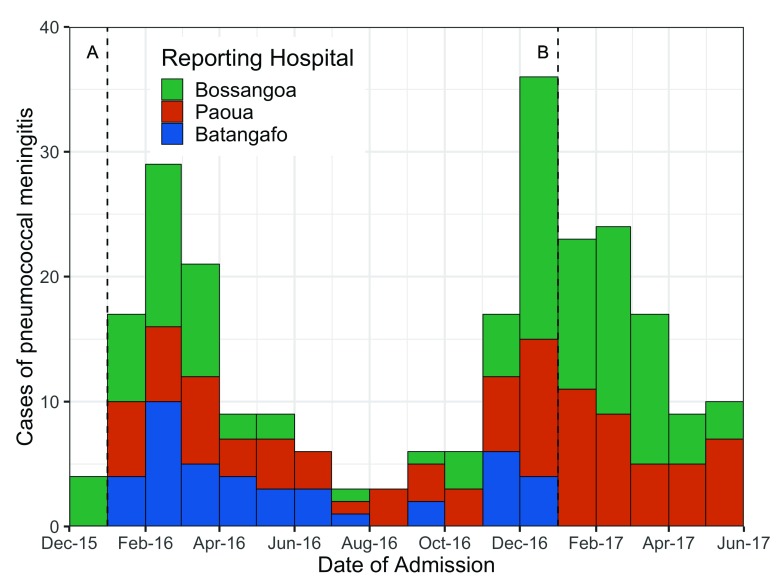
Cases of confirmed pneumococcal meningitis from three hospitals in the Central African Republic from 1st December 2015 to 31st May 2017. The plot shows a histogram where cases are collected into bins of one month. Cases are more numerous during the dry season (November to April), suggesting a seasonal trend. The dashed line A denotes the start of the reporting period for the health facilities in Batangafo and Paoua and B denotes the end of the reporting period for Batangafo. Bins are coloured by hospital.

**Figure 3.**
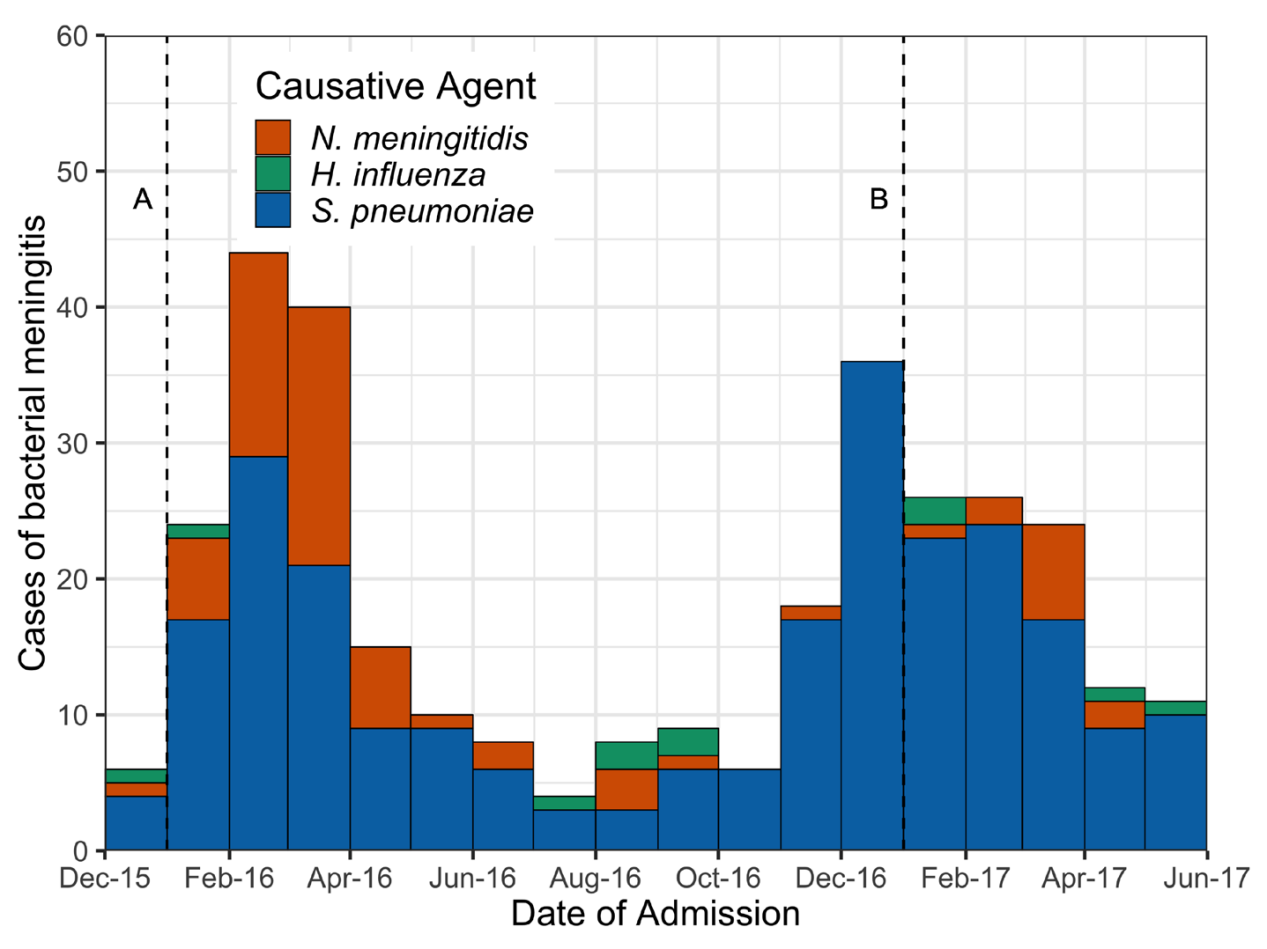
Cases of confirmed bacterial meningitis from three hospitals in the Central African Republic from 1st December 2015 to 31st May 2017. The plot shows a histogram where cases are collected into bins of one month. Cases are more numerous during the dry season (November to April), suggesting a seasonal trend. The dashed line A denotes the start of the reporting period for the health facilities in Batangafo and Paoua and B denotes the end of the reporting period for Batangafo. Bins are coloured by causative agent ( *Neisseria meningitidis*, *Haemophilus influenzae*, *Streptococcus pneumoniae*).

In Bossangoa Hospital, 139 cases of confirmed bacterial meningitis were reported over 18 months (Dec 2015 – end May 2017). The median case age was 16 years (Interquartile range [IQR] 7, 32) and 52.2% of patients were male. The majority of confirmed cases were caused by *Streptococcus pneumoniae* (pneumococcal meningitis); 114/139 (82.0%), rather by than *Neisseria meningitidis* (meningococcal meningitis) and this was also observed in the majority of confirmed meningitis cases in Paoua Hospital over 17 months (93/106, 87.7%) and Batangafo Hospital over 12 months (42/83, 50.6%), see Table 1 and Figure 2 for cases of pneumococcal meningitis by reporting Hospital. Across all sites, the majority of confirmed cases of bacterial meningitis were caused by *S*. *pneumoniae* (249/328; 75.9%) followed by *N*. *meningitidis* (67/328; 20.4%) and *Haemophilus influenzae* (12/328; 3.7%); see Figure 3 for cases by causative agent over time.

**Table 1.** Summary of the patients diagnosed as positive for bacterial meningitis from three hospitals in the northwest Central African Republic. The number of patients with reported sex and case fatality ratios may be lower than the total numbers due to missing data. IQR, interquartile range; CFR, case fatality ratios; CI, confidence interval; pneumococcal, pneumococcal meningitis; meningococcal, meningococcal meningitis.

	Bossangoa Hospital	Batangafo Hospital	Paoua Hospital	Total
Prefecture	Ouham	Ouham	Ouham-Pendé	
Reporting Period	Dec 2015 - May 2017 (18 months)	Dec 2015 - Dec 2016 (12 months)	Jan 2016 - May 2017 (17 months)	
Total Confirmed Cases (Confirmed Pneumococcal Cases)	139 (114; 82.0%)	83 (42; 50.6%)	106 (93; 87.7%)	328 (249; 75.9%)
Median Age of Confirmed Cases (IQR)	16 years (7, 32)	10 years (5, 20)	7 years (1, 13)	11 years (4, 25)
Male Confirmed Cases (%)	72/138 (52.2%)	46/83 (55.4%)	57/105 (54.3%)	175/326 (53.7%)
Pneumococcal CFR (95% CI)	41/88 (46.6%) (36.0, 57.5%)	11/37 (29.7%) (16.4, 47.2%)	Data Unavailable	52/125 (41.6%) (33.0, 50.8%)
Meningococcal CFR (95% CI)	1/15 (6.7%) (0.3, 40.0%)	4/37 (10.8%) (3.5, 40.0%)	Data Unavailable	5/52 (9.6%) (3.6, 21.8%)

There were 42 suspected cases of bacterial meningitis in Paoua Hospital over 17 months and 409 individuals that tested negative. The proportion of suspected cases relative to the total number of suspected, negative and confirmed cases (42/557; 7.5%) is low compared to other outbreaks of pneumococcal meningitis ^9^, suggesting that the majority of patients arriving with a clinical suspicion of bacterial meningitis were laboratory tested. The proportion of patients testing positive from latex agglutination test of cerebrospinal fluid (106/515; 20.6%) is comparable to a contemporaneous outbreak of pneumococcal meningitis in Ghana (23.8%) ^9^.

The temporal distribution of cases over the observed period was non-random, with the majority occurring in the dry season of November-April. Of all cases of bacterial meningitis, 175/221 (79.2%) were in the dry season months in 2016, and 128/161 (79.5%) of pneumococcal meningitis cases ( Figure 2 and Figure 3). A chi-squared ( *χ* ^2^) test for count data showed the difference between the number of cases in dry and wet season months to differ significantly for all cases of bacterial meningitis by any causative agent ( *χ* ^2^ = 75, *p* = 2.2 *×*10 ^−16^, degrees of freedom [ *df*] = 1), and for cases of pneumococcal meningitis ( *χ* ^2^ = 56, *p* = 7.0 *×* 10 ^−14^, *df* = 1). Species confirmation by PCR was consistent with the latex agglutination test for the subset of confirmed cases. Six isolates of *S. pneumoniae* from Paoua Hospital were confirmed by PCR as serotype 1, however the majority of isolates were not serotyped.

### Patient outcomes

Medical staff in MSF-supported facilities reported that patients typically presented with advanced symptoms (Glasgow Coma Scale 1-4) and that they had often sought the advice of traditional healers beforehand, though data on clinical symptoms and previous treatment at admission were not systematically recorded. Patient outcomes were recorded in Bossangoa and Batangafo ( Table 1); case fatality ratio (CFR) estimates were high for patients with *S*. *pneumoniae* in Bossangoa, 46.6% (95% confidence interval [CI] 36.0%, 57.5%) and Batangafo, 29.7% (95% CI 16.4%, 47.2%), though these values are within the range of CFR estimates for pneumococcal meningitis from other African countries in the meningitis belt (36-66%) ^10^. In Bossangoa and Batangafo the median age of pneumococcal meningitis patients was 6 years (IQR 6, 27) and 53.8% were male. In a logistic regression model where the outcome variable was death or survival, children (<16 years) admitted with pneumococcal meningitis were found to have a lower CFR (24.6% [95% CI 12.7%, 39.7%], odds ratio [OR] = 0.33, *p* = 0.0035) compared to adults, (CFR 49.2% [95% CI 33.9%, 64.6%]). A higher CFR was also found in adults with pneumococcal meningitis in Burkina Faso from 2002–05 ^11^, though age did not predict mortality in Northern Nigeria from 1971–76 ^12^. This may reflect statistical control for clinical severity in 8, and the variation in mortality we observe may be confounded by differences in healthcare-seeking behaviour between children and adults, whereby adults present later to healthcare facilities when their symptoms are more advanced ^13^. The effect of sex on the odds of mortality was not significant (OR for males = 1.39, *p* = 0.39).

### Incidence rates

The World Health Organization lacks a formal definition for outbreaks of pneumococcal meningitis ^14^; however, the ‘alert threshold’ is defined by the CAR MoH as 3 cases per 100,000 per week and ‘epidemic threshold’ as 10 cases per 100,000 per week. Bossangoa reported 11 cases of pneumococcal meningitis in the 9th epidemiological week of 2017; given an estimated target population of 350,000 this meets the alert threshold (3.1 cases per 100,000 per week; population estimate based on 2003 census).

### Meningococcal meningitis

Amongst the cases of meningococcal meningitis (67, 19.5% of total confirmed cases) the CFR estimates were significantly lower in both Bossangoa, 6.67% (95% CI 0.3, 40.0%) and Batangafo, 10.8% (95% CI 3.5, 40.0%) than those reported for pneumococcal meningitis (see Table 1). The median age of patients with meningococcal meningitis was 7 years (IQR 3, 19) and 56.1% of patients were male. Nearly all cases were group Y/ W (96.9%) with the latex agglutination test, and a subset of these were confirmed as group W by PCR at the Institute Pasteur in Bangui. This is most likely to be the strain of *N*. *meningitidis* as W is known to be present in Africa whereas strain Y is confined to North America ^15^. Recent work has shown that between 2015 and 2016 100% (66/66) of meningococcal samples isolated from patients nationwide in the CAR were of group W and that the majority of subtyped isolates belonged to the ST11 complex ^16^.

### Vaccination campaigns

A recent nationwide vaccination campaign by the CAR MoH commencing in 2016 used MenAfriVac, which is only active against meningococcal meningitis group A. While group W is considered less infectious than group A, outbreaks with W have been reported from the meningitis belt, for instance in Burkina Faso in 2002 ^17^. Vaccinations against pneumococcal meningitis have been given routinely to infants in MSF supported health facilities since 2012, and in addition MSF conducted a multi-antigen vaccination catch up campaign in the Paoua sub-prefecture for children less than 5 years from 2015 to mid-2016. This included two rounds of vaccination with pneumococcal conjugate vaccine (PCV13), which is effective against thirteen serotypes of *S*. *pneumoniae*. In the first round 70% of the population were targeted with 95% coverage. Separate attempts have been made to improve vaccine coverage in Mambéré-Kadéï prefecture in western CAR following disruption due to acute conflict ^18^. However, the coverage of PCV13 nationwide is unknown.

## Discussion and conclusion

In this study we have observed high rates of meningitis in the northern regions of the CAR from December 2015 to May 2017, predominantly caused by pneumococcus and occurring disproportionately in the dry season. A heightened incidence of bacterial meningitis during the dry season in the African meningitis belt has historically been associated with *Neisseria meningitidis*, though occasional seasonal outbreaks of *S*. *pneumoniae* have previously been observed ^11, 19^.

During our observation period from December 2015 to April 2016 a large outbreak of predominantly serotype 1 pneumococcal meningitis occurred in the Brong-Ahafo region of Ghana (886 suspected cases). The epidemiological features of this contemporaneous outbreak, including the age distribution of cases, the case fatality ratio, the proportion of aetiological agents, and the ratio of negative to positive cases, were broadly comparable to the setting we observed ^9^. Notably, this outbreak occurred despite a recent nationwide PCV13 vaccination campaign.

A subset of six pneumococcal samples were all classified as serotype 1 by PCR. While this number is small relative to the total number of samples, it confirms a previous result from the CAR which found that serotype 1 was the predominant *S. pneumoniae* serotype (44% of typed CSF samples) in a Bangui hospital from 2004-5 ^20^. Serotype 1 is among the serotypes targeted by PCV10 and PCV13. Establishing the serotype of pneumococcal meningitis during an outbreak is crucial for establishing an appropriate control strategy, such as reactive vaccination ^21, 22^.

Reliable estimates of the underlying population in the CAR are lacking which makes it challenging to calculate per-capita incidence rates. It has been estimated that one-fifth of the population of the CAR has been displaced internally or in neighbouring countries following civil conflict in 2014 ^23^. Therefore our estimates of the per-capita incidence should be treated cautiously as they likely underestimate the true rate. Improved reporting of suspected cases from Batangafo and Bossangoa would enhance epidemiological surveillance, as a threshold of 10 suspected cases per 100,000 per week has been suggested as an appropriate “epidemic threshold” and outbreak definitions using suspected cases is also employed for meningococcal meningitis ^5, 22^.

Further uncertainty surrounds the proportion of true cases that report to our medical facilities given the ongoing instability and the difficulties faced by patients from remote rural areas in accessing care. In February 2016 MSF medical staff made an exploratory visit to the community of Kouki in Ouham Prefecture (population 600) as a response to a rise in pneumococcal cases in Bossangoa Hospital. Community health workers reported 17 deaths with symptoms suggestive of bacterial meningitis over the previous 2 months. None of these fatal cases had reported to a healthcare facility, raising the possibility that the cases we observed represent only the ‘tip of the iceberg’ during the seasonal peak. Given the relatively low attack rate of *S*. *pneumoniae*, whereby bacteria in carriage become invasive and cause disease, it is likely that there is high underlying prevalence of *S*. *pneumoniae* carriage in the community ^24^.

In conclusion it appears that the northern region of the Central African Republic experienced an outbreak of pneumococcal meningitis, most likely serotype 1, over the observed period, with a similar seasonal pattern to meningococcal meningitis. A comprehensive follow-up of cases in the community was not possible due to security constraints. Despite a MenAfriVac campaign conducted in 2017 meningococcal meningitis is still present although with predominantly non-A groups circulating, namely W. Our analysis is limited by incomplete PCR confirmation and serotyping for *S*. *pneumoniae*, however our findings suggest that increasing PCV13 coverage in routine vaccination programmes would be beneficial in preventing future seasonal outbreaks of pneumococcal meningitis.

## Data availability

Raw, de-identified data taken from the present study are available on figshare, DOI: https://dx.doi.org/10.6084/m9.figshare.7210367 ^25^.

Data are available under the terms of the https://creativecommons.org/licenses/by/4.0/ (CC-BY 4.0).
