# Supplementary material for: Seasonal upsurge of pneumococcal meningitis in the Central African Republic
Source: Wellcome Open Res. 2019 Mar 29;3:134. Originally published 2018 Oct 19. [Version 2] doi: 10.12688/wellcomeopenres.14868.2 (PMC6480959; doi:10.12688/wellcomeopenres.14868.2)
Supplement: Peer review history

# Peer review: Reviewer response for version 2

**DOI:** 10.21956/wellcomeopenres.16584.r35167

**Published:** 2019-05-15

**Authors:** Caroline Trotter

**Affiliations:** 1 Department of Veterinary Medicine, University of Cambridge, Cambridge, UK

Thank you for addressing my previous comments.

I have read this submission. I believe that I have an appropriate level of expertise to confirm that it is of an acceptable scientific standard.

# Peer review: Reviewer response for version 2

**DOI:** 10.21956/wellcomeopenres.16584.r35166

**Published:** 2019-05-09

**Authors:** Hélène Broutin

**Affiliations:** 1 Department of Parasitology, Faculty of Medicine, Cheikh Anta Diop University (UCAD), Dakar, Senegal; 2 MIVEGEC, UMR CNRS, IRD, University of Montpellier, Montpellier, France

I think that authors replied and amended the paper accordingly to my first comments. I would accept this new version for indexing.

I have read this submission. I believe that I have an appropriate level of expertise to confirm that it is of an acceptable scientific standard.

# Peer review: Reviewer response for version 2

**DOI:** 10.21956/wellcomeopenres.16584.r35168

**Published:** 2019-04-23

**Authors:** Alexandre Manirakiza

**Affiliations:** 1 Epidemiological Service, Pasteur Institute of Bangui, Bangui, Central African Republic

I feel the authors provided satisfactory information to my previous comments. So, I have no further comments.

I have read this submission. I believe that I have an appropriate level of expertise to confirm that it is of an acceptable scientific standard.

# Peer review: Reviewer response for version 1

**DOI:** 10.21956/wellcomeopenres.16204.r34726

**Published:** 2019-02-11

**Authors:** Alexandre Manirakiza

**Affiliations:** 1 Epidemiological Service, Pasteur Institute of Bangui, Bangui, Central African Republic

This article reports very important information for understanding the epidemiology of meningitis in the Central African Republic.

 However, some additional information can be brought to the article for the reader.

1. Introduction: The introduction could be more informative about the context of this work if the authors add some bibliographical references, such as the existing and published data on bacterial meningitis in the Central African Republic ^1^^-^^3^.
2. Methods/Patients: How many patients where excluded? Why were suspected cases excluded? These cases were excluded because they had a negative result or because cerebrospinal fluid analysis could not be performed? Details about this, should be provided at the beginning of the Result section.
3. Statistical analysis: Authors stated that “Patient data were recorded using Microsoft Excel”. They should provide these data in their Excel format. Visibly, this is not the case at this link: https://dx.doi.org/10.6084/m9.figshare.7210367.
4. Results/Discussion: It seems to me more interesting to separate these two parts.
5.  Conclusion: The limitations of this study  (“A comprehensive follow-up of cases in the community….. Our analysis is limited by incomplete PCR confirmation and a lack of serotyping……”could be moved at the end of the discussion.

I have read this submission. I believe that I have an appropriate level of expertise to confirm that it is of an acceptable scientific standard, however I have significant reservations, as outlined above.

## References

[1] ColdironMETouréOFrankTBouyguesNGraisRF: Outbreak of Pneumococcal Meningitis, Paoua Subprefecture, Central African Republic, 2016-2017. *Emerg Infect Dis*.24(9) : 10.3201/eid2409.171058 1720-1722 10.3201/eid2409.171058 30124413PMC6106441

[2] BercionRBobossi-SerengbeGGodyJCBeyamENManirakizaALe FaouA: Acute bacterial meningitis at the 'Complexe Pédiatrique' of Bangui, Central African Republic. *J Trop Pediatr*.2008;54(2) : 10.1093/tropej/fmm075 125-8 10.1093/tropej/fmm075 17906317

[3] Cerebrospinal meningitis in the Central African Republic. *Wkly Epidemiol Rec*.1992;67(45) :333-5 1419569

# Response

**Published:** 2019-03-20

**Authors:** Tom C

We thank Dr. Manirakiza for his helpful comments.

To address the specific issues he raises:

1. Introduction: We have expanded the introduction and the discussion/ conclusion to place the current study in a broader context, including incorporating the recommended references. 

2. Methods/Patients: Data on patients that tested negative and suspected cases were collected only in Paoua Hospital. We have added a paragraph describing the suspected and negative cases in the methods section. We are aware of this limitation in our data from Batangafo and Bossangoa and encourage more thorough reporting of suspected and negative cases in the future. 

3. Statistical analysis: The data we provide at https://dx.doi.org/10.6084/m9.figshare.7210367 is a .txt file which is readable by Excel. It can be imported by (File -> Import -> Text file).

4. Results/Discussion: We have added a discussion section where we discuss the study in a broader context. We retain some discussion elements in results sections as we feel that this contributes better to reader flow and prevents the discussion / conclusion section from being overly repetitive.  

5. Conclusion: We have added a discussion section as mentioned above.

In addition to these points we have added information regarding serotyping of pneumococcus and added details of other outbreaks of pneumococcal meningitis that occurred at a similar time.

Overall we consider the quality and usefulness of our article to be substantially improved in light of the comments by Dr. Manirakiza and the other reviewers.

# Peer review: Reviewer response for version 1

**DOI:** 10.21956/wellcomeopenres.16204.r34638

**Published:** 2019-01-31

**Authors:** Hélène Broutin

**Affiliations:** 1 Department of Parasitology, Faculty of Medicine, Cheikh Anta Diop University (UCAD), Dakar, Senegal; 2 MIVEGEC, UMR CNRS, IRD, University of Montpellier, Montpellier, France

This study represents an important contribution to understand the current epidemiology situation of bacterial meningitis in the African Belt in the context of One serogroup targeted vaccine. Pneumococcal meningitis continues to kill people and this contribution could help in designing potential new vaccination strategies for meningitis control.

 The limit of this study consists in the lack of information about the serotypes of Streptococcus pneumonia. Indeed, authors conclude from their results that PCV vaccines can help to control pneumo meningitis control but they did not provide serotypes information for cases in comparison to serotypes included in PCV vaccine. The study deserves to be published without this information about serotypes but authors should discuss this lack of serotype information as a limitation and explain that it should be a next step of the study to be able to inform the potential role of PCV vaccination in the meningitis control. 

 The paper is simply and clearly written. Introduction could be more developed to provide readers with a brief overview of what is known about pneumo meningitis in the African belt. The point is that this information is lacking so far and that makes this study highly relevant and informative.

I have read this submission. I believe that I have an appropriate level of expertise to confirm that it is of an acceptable scientific standard, however I have significant reservations, as outlined above.

# Response

**Published:** 2019-03-20

**Authors:** Tom C

We thank Dr Broutin for her helpful remarks.

We have added details on serotyping. Six pneumococcal CSF isolates have been typed as serotype 1 by PCR. While this represents a small proportion of the total isolates, it confirms previous research in the Central African Republic that found serotype 1 to be the dominant strain in *S. pneumoniae *meningitis cases in Bangui. An outbreak that occurred contemporaneously in Ghana in 2015/6 was also predominantly pneumococcus serotype 1. This suggests that PCV10/13 would be effective against the outbreak strains of *S. pneumoniae *in CAR.

We have also expanded the introduction and discussion to place the CAR outbreak in a broader context and included more references.

Overall we consider the article to be substantially improved and more informative after the comments from Dr Broutin and other reviewers.

# Peer review: Reviewer response for version 1

**DOI:** 10.21956/wellcomeopenres.16204.r34111

**Published:** 2018-11-15

**Authors:** Caroline Trotter

**Affiliations:** 1 Department of Veterinary Medicine, University of Cambridge, Cambridge, UK

This is an interesting paper describing the epidemiology of bacterial meningitis in CAR. While this is clearly presented, I have two major comments about suspect vs confirmed cases and references to the current literature.

 1. The analysis concentrates on laboratory confirmed cases, which contains the richest information. Suspect cases were excluded. However, many more suspect cases than laboratory confirmed cases are identified in enhanced meningitis surveillance, which are collated and reported weekly by WHO-IST (see amongst others Lingani *et al.* ^1^. It would therefore be very useful to also report the patterns of suspected cases. What proportion of suspect cases were laboratory confirmed? What proportion of CSFs tested were negative? What was the CFR of the suspect cases? (high CFR might imply they were also likely to be due to Spn). Does the incidence of suspected cases exceed alert and epidemic thresholds? I think this would provide important context for the more detailed description of lab confirmed cases presented currently.

 2. An outbreak of Spn meningitis occurred in Ghana in 2016 - see Kwambana-Adams *et al*. ^2^ - and this paper should be considered and referenced. For example, this outbreak occurred in the context of high uptake with infant PCV. Further published work has also examined the need for alternative routine and reactive vaccination programmes in the context of Spn outbreaks ^3^^-^^4^.

 Other minor points:

 - Please use meningococcal meningitis group A (not type A)

 - In the abstract there is speculation about Spn carriage. It is indeed likely high, but this will be true in many sub-Saharan African settings. Probably more important than prevalence is distribution of serotypes, and in the absence of serotype information on cases, I don't feel that this comment on carriage adds much. It is more important to highlight the information on disease burden outside of the reporting hospitals.

 - Did you refer to the 2014 WHO epidemic meningitis guidelines?

I have read this submission. I believe that I have an appropriate level of expertise to confirm that it is of an acceptable scientific standard, however I have significant reservations, as outlined above.

## References

[1] LinganiCBergeron-CaronCStuartJMFernandezKDjingareyMHRonveauxOSchnitzlerJCPereaWA: Meningococcal Meningitis Surveillance in the African Meningitis Belt, 2004-2013. *Clin Infect Dis*.2015;61 Suppl 5: 10.1093/cid/civ597 S410-5 10.1093/cid/civ597 26553668PMC4639499

[2] Kwambana-AdamsBAsiedu-BekoeFSarkodieBAfrehOKumaGOwusu-OkyereGFoster-NyarkoEOheneSOkotCWorwuiAOkoiCSenghoreMOtuJEbrukeCBannermanRAmponsa-AchianoKOpareDKayGLetsaTKaluwaOAppiah-DenkyiraEBampoeVZamanSPallenMD’AlessandroUMwendaJAntonioM: An outbreak of pneumococcal meningitis among older children (≥5 years) and adults after the implementation of an infant vaccination programme with the 13-valent pneumococcal conjugate vaccine in Ghana. *BMC Infectious Diseases*.2016;16(1) : 10.1186/s12879-016-1914-3 10.1186/s12879-016-1914-3 27756235PMC5070171

[3] StuartJ: Can infant vaccination prevent pneumococcal meningitis outbreaks in sub-Saharan Africa?. *Tropical Medicine & International Health*.2017;22(5) : 10.1111/tmi.12860 514-515 10.1111/tmi.12860 28244176

[4] CooperLVStuartJMOkotCAsiedu-BekoeFAfrehOKFernandezKRonveauxOTrotterCL: Reactive vaccination as a control strategy for pneumococcal meningitis outbreaks in the African meningitis belt: Analysis of outbreak data from Ghana. *Vaccine*.2018; 10.1016/j.vaccine.2017.12.069 10.1016/j.vaccine.2017.12.069 29371015

# Response

**Published:** 2019-03-20

**Authors:** Tom C

In response to the helpful comments by Prof. Trotter we have included information on suspected and negative cases (this was possible in only one of the reporting hospitals), and we have expanded the introduction and discussion section to place the outbreak in CAR into a broader context. We include all of the suggested references. We discuss the contemporaneous outbreak of pneumococcal meningitis in Ghana in the discussion. We also include brief information on serotype (six pneumococcus isolates were typed by PCR as serotype 1). Overall we consider the quality and usefulness of the article has improved substantially as a result of the comments by Prof Trotter and the other reviewers.
